# Molybdenum-based nanoclusters act as antioxidants and ameliorate acute kidney injury in mice

**DOI:** 10.1038/s41467-018-07890-8

**Published:** 2018-12-21

**Authors:** Dalong Ni, Dawei Jiang, Christopher J. Kutyreff, Jianhao Lai, Yongjun Yan, Todd E. Barnhart, Bo Yu, Hyung-Jun Im, Lei Kang, Steve Y. Cho, Zhaofei Liu, Peng Huang, Jonathan W. Engle, Weibo Cai

**Affiliations:** 1 Departments of Radiology and Medical Physics, University of Wisconsin, Madison, WI 53705 USA; 2 Guangdong Key Laboratory for Biomedical Measurements and Ultrasound Imaging, School of Biomedical Engineering, Shenzhen University, 518060 Shenzhen, China; 3 Medical Isotopes Research Center and Department of Radiation Medicine, School of Basic Medical Sciences, Peking University Health Science Center, 100191 Beijing, China; 4 University of Wisconsin Carbone Cancer Center, Madison, WI 53705 USA

## Abstract

Acute kidney injury (AKI) is a common reactive oxygen species (ROS)-related renal disease that causes numerous deaths annually, yet only supportive treatment is currently available in the clinics. Development of antioxidants with high accumulation rates in kidneys is highly desired to help prevent AKI. Here we report molybdenum-based polyoxometalate (POM) nanoclusters with preferential renal uptake as novel nano-antioxidants for kidney protection. These POM nanoclusters, with a readily variable valence state of molybdenum ions, possess the capability to scavenge detrimental ROS. Our results demonstrate that POM nanoclusters can efficiently alleviate clinical symptoms in mice subjected to AKI, as verified by dynamic PET imaging with ^68^Ga-EDTA, serum tests, kidney tissue staining, and biomarkers detection in the kidneys. The protective effect of POM nanoclusters against AKI in living animals suggests exploring their use for the treatment of AKI patients, as well as patients with other ROS-related diseases.

## Abstract (web summary)

There are currently no effective therapies available for acute kidney injury (AKI). Here the authors generate molybdenum-based polyoxometalate nanoclusters and show that these have preferential renal uptake and can ameliorate AKI pathology in mice by scavenging reactive oxygen species.

## Introduction

Acute kidney injury (AKI), previously known as acute renal failure, has been involved in more than 5000 cases per million people and led to more than 1.7 million deaths per year^1,2^. AKI refers to clinical manifestation characterized by a rapid decrease of renal excretory function with the decreased urine output and increased accumulation of nitrogen metabolism^3^. The reactive oxygen species (ROS) that react with lipids, nucleic acids, and proteins to trigger oxidative stress and inflammation, are closely related to AKI in many cases^4,5^. Small amounts of ROS produced during renal oxidative metabolism in healthy kidneys are tolerated without any apparent side effects. However, when such a process is aberrant, ROS generated in excess by renal-infiltrating or endogenous cells may trigger the damaging of the kidneys and caused AKI^6^. Since no specific therapies have emerged, except renal dialysis and transplantation^7,8^, ROS are one of the critical targets in the prevention of AKI^9,10^. The antioxidant *N*-acetyl cysteine (NAC) has shown some success in the prevention of contrast-induced AKI according to previous reports^11,12^. Since then, numerous follow-up studies have been conducted to investigate the benefit of NAC in the prevention of AKI, but leaving clinicians uncertain of the benefit of this drug^13,14^.

Recent advances in nanomedicine have enabled innovative treatment of ROS-related diseases using various functional nanomaterials^15,16^, such as carbon^17,18^, platinum^19^, ceria^20,21^, redox polymer^22^, melanin nanoparticles (NPs)^23^, and so on. However, most of these NPs with their large size are nonspecifically uptaken by mononuclear phagocyte systems (e.g., liver and spleen), which are not subject to renal uptake due to the glomerular filtration barrier^24^. Ultra-small NPs (e.g., Cornell dots, quantum dots, Au NPs, etc.) with a hydrodynamic diameter falling below the kidney filtration threshold can pass through the glomerulus and are excreted within the certain time scale^25–30^, but most of them are not antioxidants. Nagasaki and co-workers designed pH-responsive nitroxide radical-containing NPs to act as ROS scavengers, leading to efficient relief of AKI^31^. Although the side effect of nitroxide radicals was suppressed by compartmentalizing the nitroxide radicals into the core nanostructures, the local release of nitroxide radical in untargeted area could cause damage to the normal tissue/organs^32^, and the systematic biocompatibility of these toxic derivatives-containing NPs still remains a concern. Rational design and synthesis of novel antioxidative nanomaterials with high renal uptake and low toxicity in vivo to efficiently scavenge ROS is of great significance in the prevention of AKI.

As smart, self-adaptive nano-theranostics, molybdenum (Mo)-based polyoxometalate clusters (POM) with ultra-small size have shown high rates of accumulation in the kidneys over a long period of time (>24 h) in our previous findings^33^. Moreover, the Mo ions in POM exhibited readily variable valence state between Mo^5+^ and Mo^6+^ under certain reduced or oxidized conditions^34^. Qu and co-workers found that transition-metal-substituted POM could act as functional anti-amyloid agents for Alzheimer’s disease^35^. In our previous research, the Mo-based POM clusters were radiolabeled with ^89^Zr, which allowed for the efficient staging of kidney dysfunction by in vivo positron emission tomography (PET) imaging^36^. However, evaluating kidney disease is not the final goal and the development of specific therapy for kidney diseases is an unmet clinical demand.

**Fig. 1:**
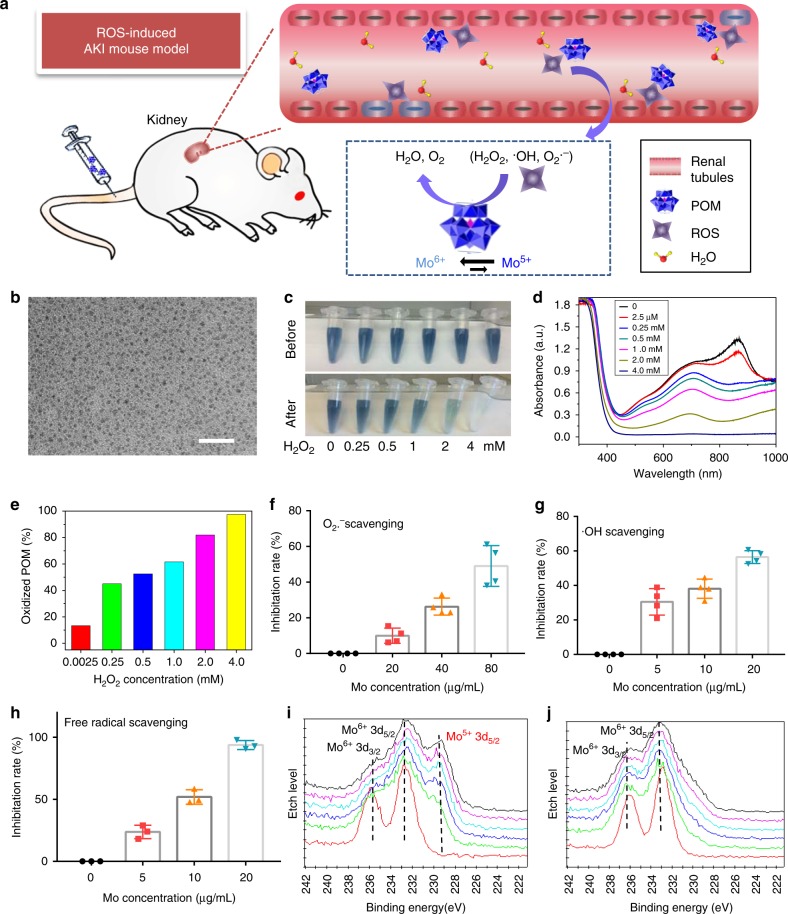
Schematic of AKI treatment using POM nanoclusters and characterization. **a** Schematic illustration of POM nanoclusters in prevention of AKI. **b** TEM image of POM at pH = 7.4; Scale bar: 20 nm. Photographs (**c**) and UV–vis–NIR spectra (**d**) of POM before and after incubation with H_2_O_2_ at different concentrations. **e** Percent oxidized POM calculated from the absorbance peak in **d**. O_2_^•−^ (**f**), ^•^OH (**g**), and free radical (**h**)-scavenging activity of POM at different concentrations. X-ray photoelectron spectroscopy of POM nanoclusters before (**i**) and after (**j**) oxidizing by the ROS. In **f**–**h**, data represent means ± s.d. from three (**h**) or four (**f**, and **g**) independent replicates

Herein, we report the capability of POM nanoclusters as antioxidants in the prevention of AKI induced by ROS in the murine model (Fig. 1a). Both in vitro and in vivo experiments show that POM nanoclusters exhibit remarkable ROS-scavenging activity by shifting between the reduced and oxidized forms. The therapeutic effect of nanoclusters against AKI may open up new perspectives for the development of nanotechnology in the treatment of AKI.

## Results and Discussion

### Characterization of POM nanoclusters

The Mo-based POM nanoclusters were synthesized via our previously reported procedure with little modification; the procedure is a fast, low-cost, and large-scale method (Supplementary Figure 1)^33^. Transmission electron microscopy (TEM) image showed that these blue POM nanoclusters were uniform with an average diameter of ca. 1 nm (Fig. 1b), and all expected essential chemical elements (Mo, O, and P) were validated by the energy dispersive X-ray (EDX) spectrum (Supplementary Figure 2). As shown in Supplementary Figure 3a, the hydrated size of POM is below 10 nm, which meets the threshold for kidney filtration and thus are able to pass through. The POM nanoclusters are highly hydrophilic and can be well dispersed in water or PBS (Supplementary Figure 3b), showing high concentration-dependent absorption in the near-infrared region in the UV–vis–NIR spectra (Supplementary Figure 4), the behavior at the origin of which was attributed to the charge transfer between Mo^5+^ and Mo^6+^ through the bridging oxygen bonds^37^. Therefore, the valence change between Mo^5+^ and Mo^6+^ can be easily monitored through the UV–vis–NIR spectrum, where increased Mo^6+^/Mo^5+^ ratio leads to decreased absorption^33,34^.

### Multi-antioxidative activities of POM nanoclusters

Three representative ROS, H_2_O_2_, O_2_^•^^−^, and ^•^OH, were selected to investigate the ROS-scavenging activity of POM nanoclusters at different concentrations. As shown in Fig. 1c, the POM exhibited high H_2_O_2_ scavenging activity, and the blue color of POM became weaker with increasing H_2_O_2_ concentration from 0.25 mM to up 4 mM; the weakening of the blue color was attributed to the readily oxidized Mo^5+^ by H_2_O_2_, which resulted in decreased NIR absorption (Fig. 1d and Supplementary Figure 5). It should be noted that the POMs are very sensitive to H_2_O_2_, even the scavenging of 2.5 μM H_2_O_2_ can be easily detected through the decreased NIR absorption in the UV–vis–NIR spectrum (Fig. 1d, e). The antioxidative activity of POM toward O_2_^•−^ and ^•^OH were also examined, and both assays showed the highly sensitive and concentration-dependent scavenging of O_2_^•−^ and ^•^OH (Fig. 1f, g), indicating that these POMs were robust scavengers of O_2_^•−^ and ^•^OH. To further confirm the antioxidative properties of POM, the free radical scavenging of POM was performed by the widely used ABTS (2,2′-azinobis (3-ethylbenzothiazoline 6-sulfonate)) radical assay. As shown in Fig. 1h and Supplementary Figure 6, more than 90% of the free radicals were eliminated by the POM at a very low concentration (20 μg Mo/mL). Similar to the mechanism of ceria nanoparticles^38^, the high ROS-scavenging performance of POM is ostensibly attributed to the fact that part of Mo^5+^ in POM is oxidized to Mo^6+^ state when eliminating the ROS, which has further been confirmed by X-ray photoelectron spectroscopy (Fig. 1i, j)^33^.

### Scavenging ROS with POM nanoclusters in vitro

**Fig. 2:**
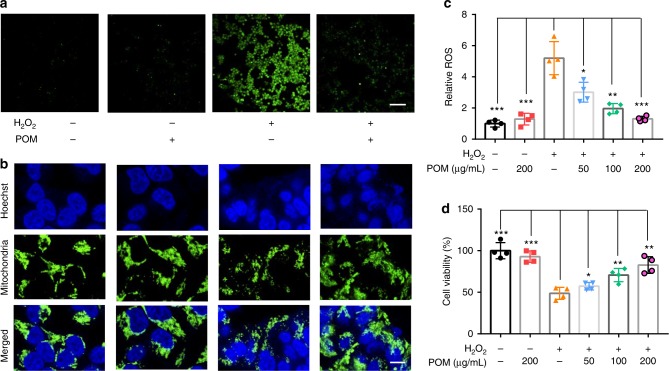
Scavenging ROS with POM nanoclusters in vitro. **a** Representative ROS staining of HEK293 cells under different treatment conditions. Scale bar: 50 μm. **b** Representative mitochondria staining of HEK293 cells under different treatment conditions. Scale bar: 5 μm. **c** ROS levels in untreated and POM-treated HEK293 cells incubated with 0.25 mM H_2_O_2_. **d** In vitro cell viabilities of HEK293 cells under different treatment conditions. In **c** and **d**, data represent means ± s.d. from four independent replicates, and *P* values were calculated by two-tailed Student’s *t*-test (*P < 0.05; **P < 0.01; ***P < 0.001)

Given the strong antioxidative property of POM nanoclusters, we proceeded to examine their potential capability in scavenging ROS in vitro. As renal tubules are vulnerable to damage by oxidative stress, we first used human embryonic kidney 293 (HEK293) cells to evaluate the protective effects of POM nanoclusters against ROS. The result of MTT showed that no obvious cytotoxicity of POM nanoclusters was found even under a high POM concentration (Supplementary Figure 7). Comparing with the control group, HEK293 cells with POM treatment did not produce excess ROS or affect mitochondrial function (Fig. 2a, b). However, intracellular ROS was dramatically enhanced upon H_2_O_2_ treatment (Fig. 2a), and significant mitochondrial fragmentation was found concurrently, which is consistent with the previous result that overproduction of ROS will induce mitochondrial damage^39,40^. By comparison, the cells pre-treated with POM nanoclusters experienced a significant drop in ROS concentration upon H_2_O_2_ treatment, indicating the scavenging of ROS by nanoclusters, and no obvious mitochondrial fragmentation was found (Fig. 2b). Further quantitative analysis of ROS levels in cells showed that the presence of POM could reduce ROS to a certain extent, depending on the POM concentrations (Fig. 2c). In addition, high ROS levels in HEK293 cells could destroy the mitochondrial function and cause cell death as a result of oxidative stress, whereas POM nanoclusters clearly reduced such response and protected the cells from destruction by ROS (Fig. 2d).

### Biodistribution of POM nanoclusters in AKI mice

**Fig. 3:**
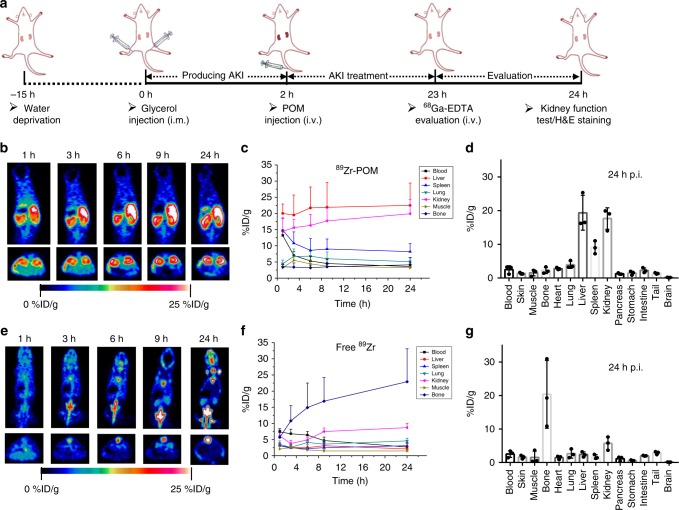
Schematic of AKI treatment and biodistribution of POM nanoclusters in AKI mice. **a** Preparation and treatment schedule of AKI mice. Representative longitudinal PET imaging of ^89^Zr-POM (**b**) and free ^89^Zr (**e**) in mice with AKI. (top: coronal slices; down: axial slices). Quantification of ^89^Zr-POM (**c**) and free ^89^Zr (**f**) uptake in the blood, liver, spleen, kidneys, and muscle at various time points p.i. Biodistribution of ^89^Zr-POM (**d**) and free ^89^Zr (**g**) at 24 h p.i. in AKI mice as determined by ^89^Zr radioactivity measurement in various tissues and organs. In **c**, **d** and **f**, **g**, data represent means ± s.d. from three independent replicates

Encouraged by in vitro results, we first evaluated the biodistribution of POM nanoclusters in AKI mice via the powerful positron emission tomography (PET) imaging. The highly oxophilic radionuclide ^89^Zr can be easily labeled with the oxygen-rich POM nanoclusters^41,42^, and the labeling yield herein was measured to be as high as 92.6% (Supplementary Figure 8a). These ^89^Zr-POM nanoclusters were highly stable in PBS and blood serum as monitored by thin layer chromatography, where more than 90% of ^89^Zr-POM was remained intact within 24 h (Supplementary Figures 8b–9). The framework, size, and behavior of ^89^Zr-POM remained the same^36^, which was reasonably expected due to the extremely little amount of ^89^Zr (<1 nmol/mCi) used for radiolabeling. In the meantime, the murine model of AKI was built through intramuscular injection of 50% glycerol into dehydrated normal mice (Fig. 3a)^43,44^. Two hours after induction of AKI, ^89^Zr-POM was intravenously injected into AKI mice. Free ^89^Zr radioisotope was intravenously injected into another group of AKI mice as a control. Longitudinal PET imaging was then performed at various time points to monitor the behavior of the tracer in vivo.

As shown in Fig. 3b, c, ^89^Zr-POM nanoclusters showed high and gradual accumulation in the injured kidneys at all examined time points, which was attributed to the ultra-small hydrodynamic diameter of POM nanoclusters (Supplementary Figure 3). However, non-specific uptake of ^89^Zr-POM in the liver and spleen were also found, which may be caused by serum protein adsorption of nanoclusters in biological fluid or adsorption of opsonins on ^89^Zr-POM (known as “opsonization”) in vivo^45,46^. It should be noted that the biodistribution of POM on AKI mice is a little different from the previously reported results on healthy mice^33^, which may be caused by the murine models and the different measuring method. In contrast, the kidney exhibited negligible uptake of free ^89^Zr, and high bone uptake was found in free ^89^Zr-injected AKI mice (Fig. 3e, f). This result is consistent with previous PET experiments using free ^89^Zr^42^. To further validate the accuracy of PET quantification analysis, ex vivo biodistribution studies were performed at 24 h after initiation of the AKI model. The renal uptake of ^89^Zr-POM at 24 h post-injection (p.i.) was 17.7 ± 3.1 %ID/g (Fig. 3d), which matched well with the quantification made from the PET data while only high bone uptake of free ^89^Zr was found for the control group (Fig. 3g), indicating that PET signals are derived from the intact ^89^Zr-POM.

In our previous work, the uptake of POM clusters in the kidney was found to reach a peak at 24 h p.i. and then decreased gradually. Nearly 73% ID of the POM nanoclusters were cleared through the renal system while 18% ID of POM nanoclusters were excreted through the liver metabolism in 1 week^33^. Such renal clearance behavior is very different from the classic ultra-small NPs (e.g., quantum dots), which were cleared rapidly through the kidneys after intravenous injection^27^. As smart nanoclusters, POM nanoclusters have been found to be bioresponsive to different microenvironment (e.g., pH)^33,34,47^, which complicates the renal clearance mechanism of POM nanoclusters. The Bio-TEM images of renal tissues (Supplementary Figure 10) showed the presence of POM nanoclusters (especially at 24 h p.i.) with some self-assembly^33,47^, which prolonged the renal retention of nanoclusters. It is also possible that some nanoclusters was disassembled into smaller pieces within sub-nanometer size range and bound to the glycocalyx of endothelial cells in the glomeruli, as reported by Zheng and co-workers that the glomeruli could serve as an anatomically precise barrier to slow down the renal clearance of clusters in the sub-nanometer size range^48–50^. However, it should be noted that detecting subnanomater-sized clusters in the kidneys by performing Bio-TEM imaging is still limited due to the ultra-small size and mediocre electron density of the clusters. Positive results can be obtained by performing these experiments with highly specialized training or combine with other detecting methods to reduce any false-positive interpretations. TEM imaging of the collected urine of mice after injection showed some intact POM nanoclusters (Supplementary Figure 11), verifying that they have been indeed excreted through the urine. Future efforts should be focused on investigating the detailed renal clearance mechanism of such kind of bioresponsive nanoclusters in vivo.

### Dynamic renal PET imaging of AKI mice after treatment

**Fig. 4:**
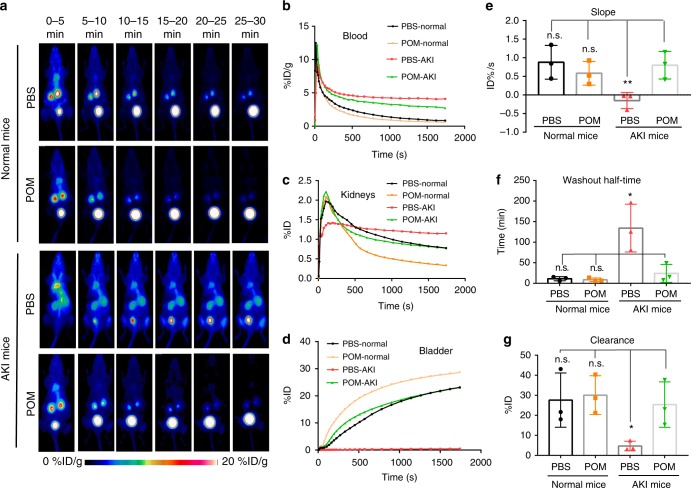
Dynamic PET imaging with ^68^Ga-EDTA tracer for evaluation of renal functions. **a** Representative PET imaging of normal mice, AKI mice treated with PBS and POM nanoclusters respectively. **b** ROI analysis of signals in the blood pool; lower signal denotes better excretion of ^68^Ga-EDTA. **c** ROI analysis of signals in the kidneys; sharper peak denotes better excretory functioning of the kidneys. **d** ROI analysis of signals in the bladder; higher signal in the bladder denotes better functioning of the kidneys. **e** The slope of renal ROI curve between 1 and 2 min p.i. of ^68^Ga-EDTA; a positive slope indicates proper functioning of the kidneys. **f** Washout half-time of renal ROI curve (time interval between the peak tracer value and half of the peak value); shorter time defines better renal excretion, further suggesting better recovery of kidney function from AKI after treatment. **g** Clearance of ^68^Ga-EDTA into the bladder; higher clearance indicates better excretion and better treatment efficacy of AKI. In **e**–**g**, data represent means ± s.d. from three independent replicates, and *P* values were calculated by two-tailed Student’s *t*-test (*P < 0.05; **P < 0.01; n.s., no significance)

The therapeutic effect was then compared between different groups, including two groups of normal mice and two groups of AKI mice, which were intravenously injected with PBS and POM nanoclusters, respectively. Dynamic PET imaging with the traditional tracer ^68^Ga-EDTA is widely used for renal imaging in the clinic^51^, allowing for real-time and non-invasive evaluation of renal function. At 23 h after the induction of AKI models, 30-min dynamic PET scans with ^68^Ga-EDTA were performed to evaluate the renal function of mice. As shown in Fig. 4a, the signal of ^68^Ga-EDTA in blood pool of normal mice decreased quickly while the signal in the bladder increased gradually after injection (Fig. 4a, d). Meanwhile, the signal in the kidney of normal mice reached the peak between 0 and 5 min and then decreased (Fig. 4c). The short remaining of ^68^Ga-EDTA in the blood pool and high accumulation in the bladder offered baseline information for both ^68^Ga-EDTA in vivo and kidney function in normal healthy mice. For AKI mice treated with PBS, sustained ^68^Ga-EDTA accumulation was found in the blood pool with little excretion of tracer to the bladder. Accumulation of the ^68^Ga-EDTA was confined and remained to the kidneys after injection, suggesting severe kidney impairment (i.e. PBS had no therapeutic effect). In contrast, AKI mice treated with POM nanoclusters showed recovery of kidney functions, where all dynamic curves of ^68^Ga-EDTA in the blood pool, kidneys, and bladder were almost the same as in the normal mice, indicating the POM nanoclusters could scavenge the ROS and produce a therapeutic effect.

Three parameters of slope, clearance, and washout half-time are commonly used in the clinic to evaluate renal function after dynamic PET imaging with ^68^Ga-EDTA tracer. Therefore, ROI analysis of renal PET images was performed to analyze these three parameters (Fig. 4e–g). The slope of the kidney ROI curves denoted a linear increase directly after injection (60 to 120 s). Slope values were significantly higher in normal mice and AKI mice treated with POM nanoclusters, while it was much lower for the AKI mice treated with PBS, suggesting the dysfunction of AKI in untreated mouse kidneys and efficient prevention of AKI by POM nanoclusters in the treated groups (Fig. 4e). Washout half-time measures the time interval between the peak renal concentration of ^68^Ga-EDTA and half of the peak value; the half-time from the normal mice groups and POM treatment groups were significantly shorter than that from the AKI control group (Fig. 4f). Tracer clearance was defined as the amount of ^68^Ga-EDTA that was effectively excreted through the kidneys to the bladder within 30 min p.i. In comparison, POM treatment groups showed a clearance of 25.4 ± 11.3 %ID (Fig. 4g), which was close to the value in normal mice (28.1 ± 13.2 %ID) or normal mice treated with POM nanoclusters (30.1 ± 9.7 %ID) and much higher than that of the AKI mice without any treatment (4.7 ± 2.3 %ID). All three parameters indicated that renal function in AKI mice was severely damaged and the POM nanoclusters played great roles in the recovery of renal function of AKI mice.

### Evaluation of AKI mice after treatment

**Fig. 5:**
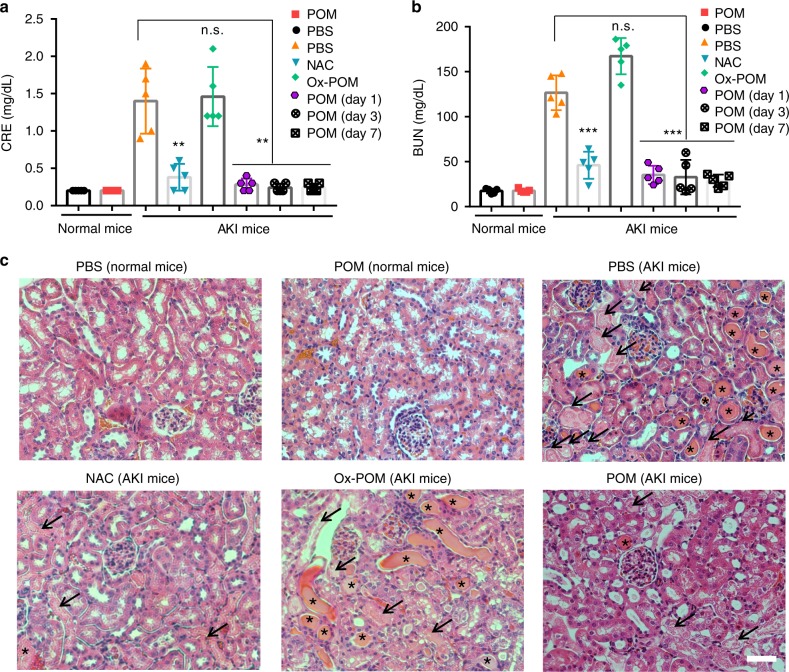
Blood serum measurements and H&E staining of renal tissue after treatment of AKI. BUN (**a**) and CRE (**b**) levels in the blood serum from each group. Lower BUN and CRE levels denote better kidney functions. **c** H&E staining of kidney tissues from each group. Arrows indicate damaged tubules, and asterisks indicate the formation of casts. Scale bar: 50 µm. In **a** and **b**, data represent means ± s.d. from five independent replicates, and *P* values were calculated by two-tailed Student’s *t*-test (**P < 0.01; ***P < 0.001; n.s. no significance)

After PET imaging with ^68^Ga-EDTA, the changes in body weight for all groups were measured, and the kidney/blood samples were then collected and analyzed for renal function at 24 h after the model preparation (Fig. 3a). In addition, to further verify the activity of POM clusters acting as ROS scavengers in vivo, the above-mentioned conventional antioxidant drug of NAC was intravenously injected into AKI mice (4 mg in 100 μL PBS, four-fold of POM nanoclusters) to use as a positive control group, while the oxidized POM (Ox-POM, mainly Mo^6+^ in the nanostructures) was used as a negative control group. Severe body weight loss in PBS or Ox-POM-treated AKI mice was observed, while the treatment of AKI with POM nanoclusters or NAC prevented weight loss (Supplementary Figure 12). Two effective clinical indices of kidney excretory function including blood urea nitrogen (BUN) and serum creatinine (CRE) levels confirmed our findings that POM nanoclusters could recover renal function in the AKI mice (Fig. 5a, b).

Hematoxylin and eosin (H&E) staining of kidney tissues was further performed to provide the direct evidence of AKI treatment. Casts are structures formed via precipitation of denatured proteins in the tubules, and are usually used as indicators for kidney diseases. As shown in Fig. 5c, both casts (marked as asterisks) and damaged renal tubules (marked as arrows) were observed in kidney sections from the PBS or Ox-POM-treated AKI mice, while much fewer damaged structures were found for POM or NAC treated groups, and no damage was found within 4 weeks after treatment of nanoclusters (Supplementary Figures 13–14). The survival curve and body weight of AKI mice were monitored after treatment for 2 weeks, which further validated the power of POM nanoclusters in the prevention of AKI (Supplementary Figure 15). To determine whether POM was effective in another AKI model, we performed this intervention on cisplatin-induced AKI (Cis-AKI) mice. As shown in Supplementary Figure 16, the damage in the kidneys from Cis-AKI mice was significantly inhibited by the treatment of POM nanoclusters in comparison to control group, suggesting that the POM nanoclusters were effective for Cis-AKI and it can address many causes of AKI.

### Biomarkers detection of AKI mice after treatment

**Fig. 6:**
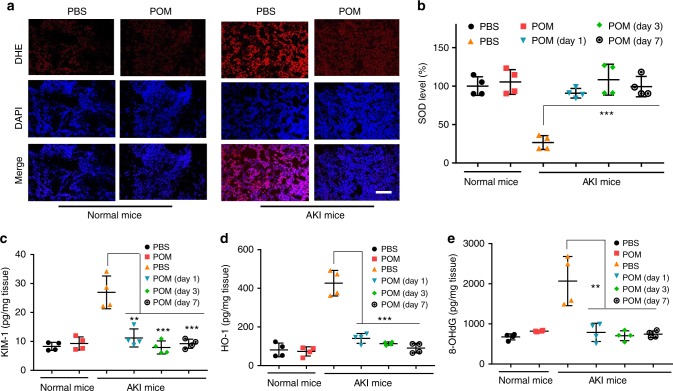
Analysis of renal tissue after treatments. **a** Dihydroethidium (DHE) and DAPI staining of kidney tissues from each group. Scale bar: 100 µm. SOD levels (**b**), KIM-1 (**c**), and HO-1 (**d**) measured in renal tissue homogenates from each group. **e** Measurement of DNA damage (8-OHdG) in renal tissue homogenates from each group. In **b**–**e**, data represent means ± s.d. from four independent replicates, and *P* values were calculated by two-tailed Student’s *t*-test (**P < 0.01; ***P < 0.001)

To further perform functional investigation of POM clusters against AKI in vivo, dihydroethidium (DHE) staining of renal tissue for superoxide production has been performed. As shown in Fig. 6a and Supplementary Figure 17, the renal generation of ROS was significantly inhibited by the treatment of POM nanoclusters. Furthermore, the level of superoxide dismutase (SOD), an important defense to neutralize ROS for nearby cells in the kidney was measured. The SOD levels in POM treatment groups at 1 day, 3 days and 7 days p.i. were similar to those of healthy mice (Fig. 6b), while reduced levels of SOD were found in PBS-treated AKI mice, indicating that POM nanoclusters served as reductants to scavenge ROS, restored SOD levels, and further protected renal cells on AKI mice. Expressions of kidney injury molecule-1 (KIM-1) and heme oxygenase-1 (HO-1) in the renal tissues, two important biomarkers of kidney injury^52,53^, were also evaluated. For POM-treating AKI mice, both KIM-1 and HO-1 were reduced to normal levels in the experimental group, close to the levels observed in healthy mice (Fig. 6c, d). Additionally, we investigated whether POM nanoclusters could inhibit DNA damage and lipid peroxidation. Compared with PBS-treated AKI mice, POM nanoclusters treatment resulted in a significant reduction of 8-hydroxy-2′-deoxyguanosine (8-OHdG) in the kidney (Fig. 6e), which served as a biomarker of oxidative stress and quantified DNA damage. A statistically significant increase of lipid peroxidation in PBS-treated AKI mice was also found, which could be inhibited by the treatment of POM nanoclusters at 1 day, 3 days and 7 days p.i. (Supplementary Figure 18). These results explain the high therapeutic efficiency of POM clusters against AKI.

### In vivo toxicity assessment

**Fig. 7:**
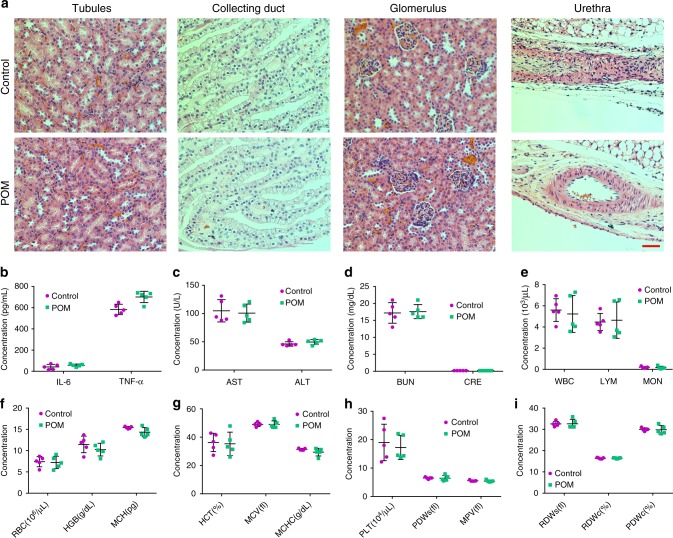
In vivo toxicity assessment of POM nanoclusters. **a** H&E-stained tissues from the kidneys. Scale bar: 50 µm. **b** Serum levels of interleukin-6 (IL-6) and tumor necrosis factor alpha (TNF-α) in normal mice (Control group) and mice injected with POM nanoclusters. **c** Serum levels of liver function indicators of alanine transaminase (ALT) and aspartate transaminase (AST). **d** kidney function indicators of blood urea nitrogen (BUN) and creatinine (CRE) in normal mice (Control group), and mice injected with POM nanoclusters. **e**–**i** Values of blood parameters in normal mice (Control group), and mice injected with POM nanoclusters. In **b**–**i**, data represent means ± s.d. from five independent replicates

To evaluate the renal toxicity of POM nanoclusters, the renal sections of tubules, collecting ducts, glomeruli, and urethrae were collected and stained with H&E p.i. of POM at 24 h, the time necessary for AKI treatment. As presented in Fig. 7a, no noticeable tissue damage or inflammatory lesions were observed when compared with the control group, suggesting that no visible lesions were formed in the kidneys after the POM treatment. Additionally, no side effects were observed in the main organs both in 1 day and 1 month (Supplementary Figures 19–20). To further and more quantitatively confirm the safety of POM, we checked the immunogenicity of POM nanoclusters by measuring blood levels of tumor necrosis factor alpha (TNF-α) and interleukin-6 (IL-6) after intravenous injection of POM and found that these nanoclusters did not cause an immune response in vivo (Fig. 7b). Liver function and kidney function of mice injected with POM nanoclusters were all within normal ranges (Fig. 7c, d). Finally, the hematology indices of mice appeared to be normal when compared with the control group (Fig. 7e–i). The above results suggest that the POM nanoclusters display excellent compatibility and therefore are promising nano-antioxidants for AKI treatment.

In summary, we presented here a novel multi-antioxidant in the form of POM nanoclusters for antioxidative therapy of AKI in living animals. Our findings revealed that POM nanoclusters exhibited broad antioxidative activities against multiple toxic ROS including H_2_O_2_, O_2_^•−^, and •OH. In vitro experiments confirmed the cells protecting activities of POM nanoclusters against harmful oxidative stress. With high renal uptake, these ultra-small nanoclusters displayed excellent therapeutic efficacy of AKI induced by ROS, which has been demonstrated by dynamic PET imaging with ^68^Ga-EDTA, CRE and BUN measurement of nitrogenous wastes, H&E staining, and biomarkers detection of kidney tissues. Therefore, the present POM nanoclusters, with excellent biocompatibility, are promising to be safe and effective nano-antioxidants for the treatment of clinical AKI and various other oxidative stress injuries.

## Methods

### Synthesis of POM nanoclusters

The POM nanoclusters were synthesized by a facile one-pot approach^33^. First, (NH_4_)_6_Mo_7_O_24_·4H_2_O (2 mmol) was dissolved in 10 mL of ultrapure water with continuous stirring at 25 °C, followed by rapidly adding 5 ml solution of 1.17 mmol of NaH_2_PO_4_. Subsequently, another 2 mL of saturated l-ascorbic acid was added dropwise into the system under stirring. For the synthesis of Ox-POM, no l-ascorbic acid was used. After continuous stirring for 15 min, the resulting POM nanoclusters were precipitated by adding 80 mL of ethanol, collected by 15 min of centrifugation at 10,304×*g*, and finally dispersed in water or PBS. To completely remove the free l-ascorbic acid, dialysis of the obtained POM nanocluster solution was performed. After being stirred for 3 days with frequent exchange with fresh solution, the final POM nanoclusters were used for further research.

### Radiolabeling of POM nanoclusters

To evaluate in vivo biodistribution of POM nanoclusters, ^89^Zr was used to label the POM nanoclusters for PET imaging. Briefly, 100 μL of POM nanoclusters dispersed in HEPES buffer was directly mixed with 1 mCi (or 37 MBq) of ^89^Zr-oxalate at 37 °C. The final pH value of the solution was adjusted to 7–8 with 1 M Na_2_CO_3_. After shaking for 2 h, ^89^Zr-POM was precipitated from the solvent by adding ethanol and collected by 15 min of centrifugation at ~20,000 × *g* and finally dispersed in PBS. ^89^Zr labeling yield was monitored and quantified by using thin layer chromatography (TLC) with subsequent autoradiography.

### Superoxide anion scavenging with POM nanoclusters

The superoxide anion scavenging activity was assessed with a SOD assay kit (Sigma-Aldrich, USA). First, 20 μL of each sample with different POM nanoclusters concentrations (0, 20, 40, and 60 μg/mL) was mixed with 160 μL of a 2-(4-iodophenyl)-3-(4-nitrophenyl)-5-(2,4-disulfophenyl)-2*H*-tetrazolium sodium salt (WST-1) working solution. Then, 20 μL of a xanthine oxidase solution was added to each microplate well. The absorbance at 450 nm was measured using a multiple plate reader after incubating at 37 °C for 20 min.

### Hydroxyl radical scavenging with POM nanoclusters

The hydroxyl radical scavenging activity was assessed using a hydroxyl radical antioxidant capacity (HORAC) assay kit (Cell Biolabs, Inc., USA). First, 20 μL of each sample with different concentrations (0, 5, 10, and 20 μg/mL) was mixed with 140 μL of fluorescent solution. Then, 20 μL of the hydroxyl radical initiator was added to each microplate. Immediately, 20 μL of Fenton reagent was added to each microplate well. After shaking for 15 s and incubating for 30 min at 25 °C, the fluorescence was measured using a multiple plate reader.

### Free radical scavenging with POM nanoclusters

The free radical scavenging capacity of POM nanoclusters was conducted using the ABTS radical cation decolorization assay, based on the reduction of ABTS+• radicals by POM nanoclusters. 7 mM ABTS was dissolved in deionized water and treated with 2.45 mM potassium persulfate to produce ABTS radical cation (ABTS+•), allowing the mixture to stay in the dark at room temperature for 24 h before use. Then, the UV–Vis spectra of pure ABTS + • solution (A_B_), and ABTS+• solution with 5, 10 and 20 μg/mL POM clusters (A_E_) were monitored to measure the absorbance. The percentage of inhibition of ABTS+• was calculated using the following equation: [(*A*_B_ − *A*_E_)/*A*_B_] × 100. All measurements were carried out in triplicate.

### XPS measurement of POM nanoclusters

X-ray photoelectron spectroscopy (XPS) measurement of POM nanoclusters was performed before and after completely oxidized by ROS (i.e., H_2_O_2_). Full scans of the nanoclusters were conducted first, then the depth profiles of spots on the samples were performed. For Ar plasma etching, we used 3000 eV for 10 s each step, for a total of five steps.

### Free radical scavenging with POM nanoclusters in cells

Human embryonic kidney 293 (HEK293) cells were purchased from the American Type Culture Collection (ATCC) and cultured under 5% CO_2_ at 37 °C in Dulbecco’s Modified Eagle Medium (DMEM) supplemented with 1% penicillin/streptomycin and 10% fetal bovine serum (FBS). The HEK293 cells were seeded into a 96-well cell culture plate at 10^4^/well and then incubated for 24 h with 5% CO_2_ at 37 °C. Culture media solutions of the POM nanoclusters with different concentrations (50, 100, and 200 μg of Mo per mL) were then added to all wells and incubated for 0.5 h. The cells were then treated with H_2_O_2_ (250 μM) and incubated for 24 h at 37 °C under 5% CO_2_. The cell wells incubated with POM nanoclusters only or H_2_O_2_ only were used as controls. Finally, the cell viability was measured by MTT assay.

To reliably measure reactive oxygen species (ROS) in live cells, a final concentration of 5 μM of fluorogenic probes (CellROX® Oxidative Stress Reagents) was added to the cells and incubated for 0.5 h at 37 °C, after the aforementioned POM nanoclusters had been treated with H_2_O_2_. Then, the cells were washed with PBS three times, and finally were measured for emission at 520 nm under excitation at 485 nm.

### Acute kidney injury (AKI) model

All animal studies were conducted under a protocol approved by the University of Wisconsin Institutional Animal Care and Use Committee. All female CD-1 ICR mice (4–6 weeks old, Envigo) were deprived of water but had access to food for 15 h. At the end of water restriction, 8 mL/kg of 50% glycerol was then intramuscularly injected into both hind limbs of mice equally, and all mice were then given free access to water and food. Symptoms of AKI, including a decrease of urine output and lack of activities, were observed within a few hours following the injections.

### Cisplatin-induced AKI model

All mice were received intraperitoneal injections of cisplatin (20 mg/kg). Either 1, 2 or 3 days after injections, the mice in each group were sacrificed to monitor the model development. The blood samples and renal tissues were obtained and analyzed. For the treatment group, the mice were intravenously injected with POM clusters (1 mg in 100 μL PBS) at 2 h after intraperitoneal injections of cisplatin.

### In vivo PET imaging of AKI mice

The AKI mice were anesthetized and intravenously injected with 150 μL (~155 μCi or 5.735 MBq) of ^89^Zr-POM in PBS (*n* = 3). Serial PET scans were performed at various time points (1, 3, 6, 9, and 24 h) post-injection (p.i.). ROI analysis of each PET scan was conducted to calculate the percentage of injected dose per gram of tissue (%ID/g) in mouse organs, using vendor software (Inveon Research Workplace [IRW]) on decay-corrected whole-body images.

After the last PET imaging at 24 h p.i., all major organs and tissues were collected and wet-weighed. The radioactivity of each tissue and organ was measured using a gamma counter and calculated as %ID/g.

### Treatment of AKI mice

Two hours after the AKI model induction, different treatments were performed on AKI model mice: group 1 was healthy mice treated with 1×PBS (*n* = 6); group 2 was healthy mice (*n* = 6) treated with POM (1 mg in 100 μl PBS), group 3 was AKI mice treated with 1×PBS (PBS, *n* = 6); group 4 was negative control group that AKI mice treated with Ox-POM (1 mg in 100 μL PBS, *n* = 6); group 5 was positive control group that AKI mice treated with NAC (4 mg in 100 μL PBS, *n* = 6); group 6 was AKI mice treated with POM (1 mg in 100 μL 1×PBS, *n* = 6). After 24 h p.i., their kidney function was compared with that of healthy control mice. The survival curve and body weight of AKI mice were monitored for 2 weeks after treatment. For a long-term assessment of renal function, another two groups of AKI mice treated with POM (*n* = 5 for each group) were killed at 3 days and 7 days respectively after injection. According to the guidelines on animal welfare, body weight loss in a mouse higher than 15% is considered to be death for the purpose of drawing the survival curve.

### Dynamic PET imaging for kidney function evaluation

To evaluate the kidney function after treatment, dynamic renal PET imaging using ^68^Ga-EDTA was performed. The mice in each group were anesthetized with 2% isoflurane and their tail veins were catheterized. A 30-min dynamic PET scan was acquired immediately after the intravenous injection of ^68^Ga-EDTA (200–500 μCi). The histogram files were framed into 28 frames: 6 × 10 s, 6 × 30 s, 6 × 60 s, and 10 × 120 s. Dynamic scans were reconstructed using an ordered subset expectation maximization 3D/maximum a posteriori (OSEM3D/MAP) reconstruction algorithm. ROI analysis of PET images was conducted to determine the time-activity curve of the blood pool, kidneys, and bladder. Tracer uptake was calculated as %ID/g for the blood pool, and %ID for the kidneys and bladder.

### Kidney function test and H&E staining of kidney sections

Kidney function test and hematoxylin and eosin (H&E) staining were performed to evaluate the treatment of AKI. Mice in all groups were killed, and blood samples were collected into pediatric heparin tubes (BD Biosciences, San Jose, CA, USA) and then centrifuged at 2000×*g* for 15 min at 4 °C to obtain the plasma. Finally, the plasma was sent to the Clinical Pathology Laboratory in Veterinary Medical Teaching Hospital at the University of Wisconsin–Madison for analysis of blood urea nitrogen (BUN) levels and blood creatinine (CRE) levels.

Kidneys were also collected 24 h after the model induction and fixed with paraformaldehyde (4% in PBS), embedded in paraffin wax and sent to the University of Wisconsin Carbone Cancer Center Experimental Pathology Laboratory for sectioning and H&E staining.

### Analysis of renal tissues after treatment

Kidneys from each group were frozen and stored at −80 °C until the assay. Kidney homogenates were prepared according to the protocols of different assays. SOD level was assessed with a SOD assay kit (Sigma-Aldrich, USA). KIM-1 expression and HO-1 expression were measured with KIM-1 or HO-1 ELISA kit (Abcam, USA). DNA damage was evaluated with DNA damage competitive ELISA kit (Invitrogen, USA). The degree of lipid peroxidation was determined with a TBARS assay kit (Cayman Chemical, USA)

### Confocal imaging of superoxide production in kidneys

To assess superoxide production histologically, kidneys harvested from mice were stored in optimum cutting temperature (O.C.T.) specimen matrix (VWR, Radnor, PA, USA) for cryostat sectioning at −20 °C. Sectioning was performed by the Experimental Pathology Laboratory at the University of Wisconsin–Madison. Frozen kidney tissue slice (about 5 μm thickness) were washed with PBS and stained with 1 mM dihydroethidium (DHE) for 30 min to detect superoxide formation. Then a cover glass was applied to each slide using Vectashield mounting medium (Vector Laboratories, Burlingame, CA, USA), and confocal imaging was performed using a Nikon A1R confocal microscope (Nikon Instruments, Melville, NY, USA).

### In vivo toxicity assessment

The POM clusters at a dose of 150 μL (2 mg/mouse) were intravenously injected into ICR mice and the mice receiving saline injection were used as the control group (*n* = 5). Organs and blood samples were harvested from two groups at 24 p.i. Organs were H&E-stained to monitor the histological changes in the heart, liver, spleen, lungs, and kidneys of the mice. The complete blood panel data from the test and control groups were measured. The relevant blood parameters were as follows: white blood cells (WBC), lymphocyte (LYM), MON, red blood cells (RBC), hemoglobin (HGB), mean corpuscular hemoglobin (MCH), hematocrit (HCT), mean corpuscular volume (MCV), mean corpuscular hemoglobin concentration (MCHC), platelets (PLT), platelet distribution width (PDW), mean platelet volume (MPV), and red cell distribution width (RDW). The blood samples were then centrifuged at 2000×*g* for 15 min at 4 °C to obtain the plasma. Finally, the plasma was sent to the Clinical Pathology Laboratory in Veterinary Medical Teaching Hospital at University of Wisconsin–Madison for analysis of two important hepatic indicators of alanine aminotransferase (ALT) and aspartate aminotransferase (AST), and two indicators for kidney functions of creatinine (CRE) and blood urea nitrogen (BUN).

### Materials

Sodium dihydrogen phosphate (NaH_2_PO_4_), hexaammonium molybdate tetrahydrate ((NH_4_)_6_Mo_7_O_24_·4H_2_O), HEPES, and l-ascorbic acid were obtained from Sigma-Aldrich. All reagents were of analytical grade and used without any purification.

### Material characterization

UV–vis spectra were recorded on an Agilent Cary 60 spectrophotometer. Dynamic light scattering (DLS) measurements were performed on a Nano-Zetesizer (Malvern Instruments Ltd). XPS measurement was performed on a Thermo Scientific K-alpha XPS. H&E staining was observed on an inverted optical microscope (Nikon, Eclipse Ti-U, Japan). The elemental concentrations (i.e., Mo) were measured by inductively coupled plasma optical emission spectrometry (ICP-OES).

## Supplementary information

Supplementary Information

## Data Availability

The authors declare that the main data supporting the findings of this study are available within the article and its Supplementary Information. Extra data that support the findings of this study are available from the corresponding authors upon reasonable request. A reporting summary for this Article is available as a Supplementary Information file.
