# Supplementary material for: Molybdenum-based nanoclusters act as antioxidants and ameliorate acute kidney injury in mice
Source: Nat Commun. 2018 Dec 21;9:5421. doi: 10.1038/s41467-018-07890-8 (PMC6303396; doi:10.1038/s41467-018-07890-8)
Supplement: Supplementary file 1 — Supplementary Information [file 41467_2018_7890_MOESM1_ESM.doc]

**Supplementary Information**

**Molybdenum-based nanoclusters act as antioxidants and ameliorate acute kidney injury in mice**

*Dalong Ni,^1^ Dawei Jiang,^1, 2^ Christopher J. Kutyreff,^1^ Jianhao Lai,^1,3^ Yongjun Yan,^1^ Todd E. Barnhart,^1^ Bo Yu,^1^ Hyung-Jun Im,^1^ Lei Kang,^1^ Steve Y. Cho**,^1^ Zhaofei Liu,^3*^ Peng Huang,^2^ Jonathan W. Engle, ^1^ and Weibo Cai,^1, 4 *^*

^1^Departments of Radiology and Medical Physics, University of Wisconsin, Madison, Wisconsin 53705, United States

^2^Guangdong Key Laboratory for Biomedical Measurements and Ultrasound Imaging, School of Biomedical Engineering, Shenzhen University, Shenzhen 518060, China

^3^Medical Isotopes Research Center and Department of Radiation Medicine, School of Basic Medical Sciences, Peking University Health Science Center, Beijing 100191, China.

^4^University of Wisconsin Carbone Cancer Center, Madison, Wisconsin 53705, United States

**Supplementary Figures**


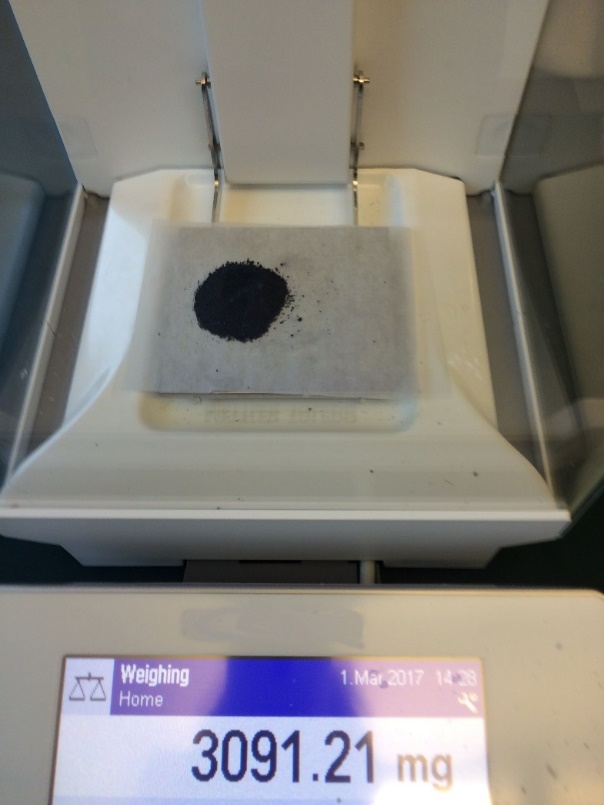


**Supplementary Figure 1.** The POM can be synthesized in a large-scale method. Photograph of the as-made POM clusters showing more than 3 g of the product, which can be further scaled up.


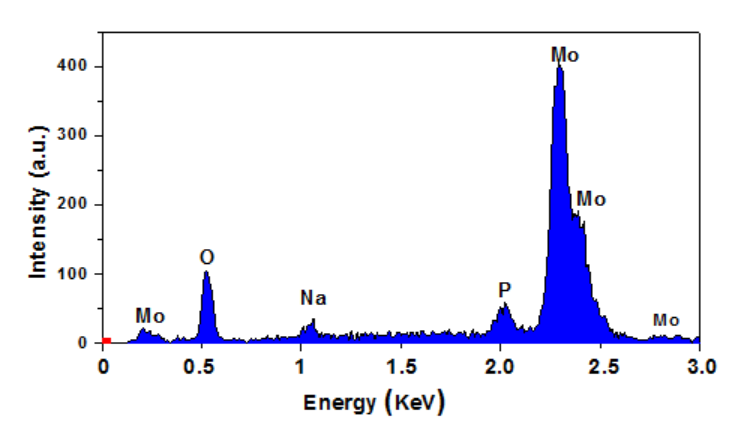


**Supplementary Figure 2.** Elements of POM nanoclusters. The energy dispersive X-ray (EDX) spectrum of the POM nanoclusters which demonstrated the existence of all the essential chemical elements (Mo, P, and O) of these clusters.


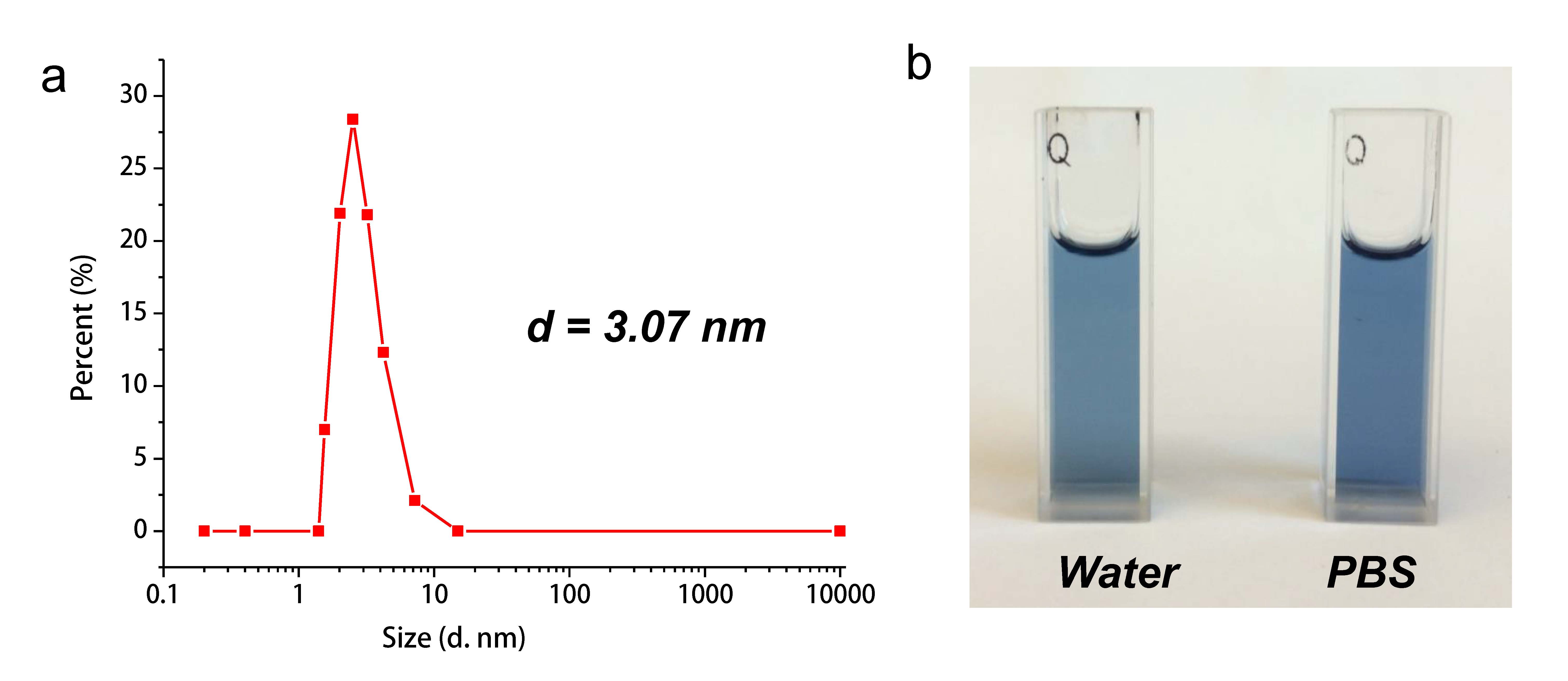


**Supplementary Figure 3.** POM nanoclusters are highly hydrophilic. a) Dynamic light scattering (DLS) measurement of the POMs dispersed in the PBS. b) Photographs of POMs dispersed in the water and PBS.

**Supplementary Figure 4.** Characterization of POM nanoclusters. UV-vis-NIR spectra of POM at various concentrations.

**Supplementary Figure 5.** POM nanoclusters scavenging H_2_O_2_. UV-vis-NIR spectra of POM before and after adding 0.25 mM H_2_O_2_.

**Supplementary Figure 6.** POM nanoclusters scavenging free radicals. UV-vis-NIR spectra of ABTS radical before and after adding POM in different concentrations.

**Supplementary Figure 7**. Cell toxicity of POM nanoclusters. *In vitro* cell viabilities of HEK293 cells incubated with different concentrations of POM nanoclusters for 24 and 48 h.


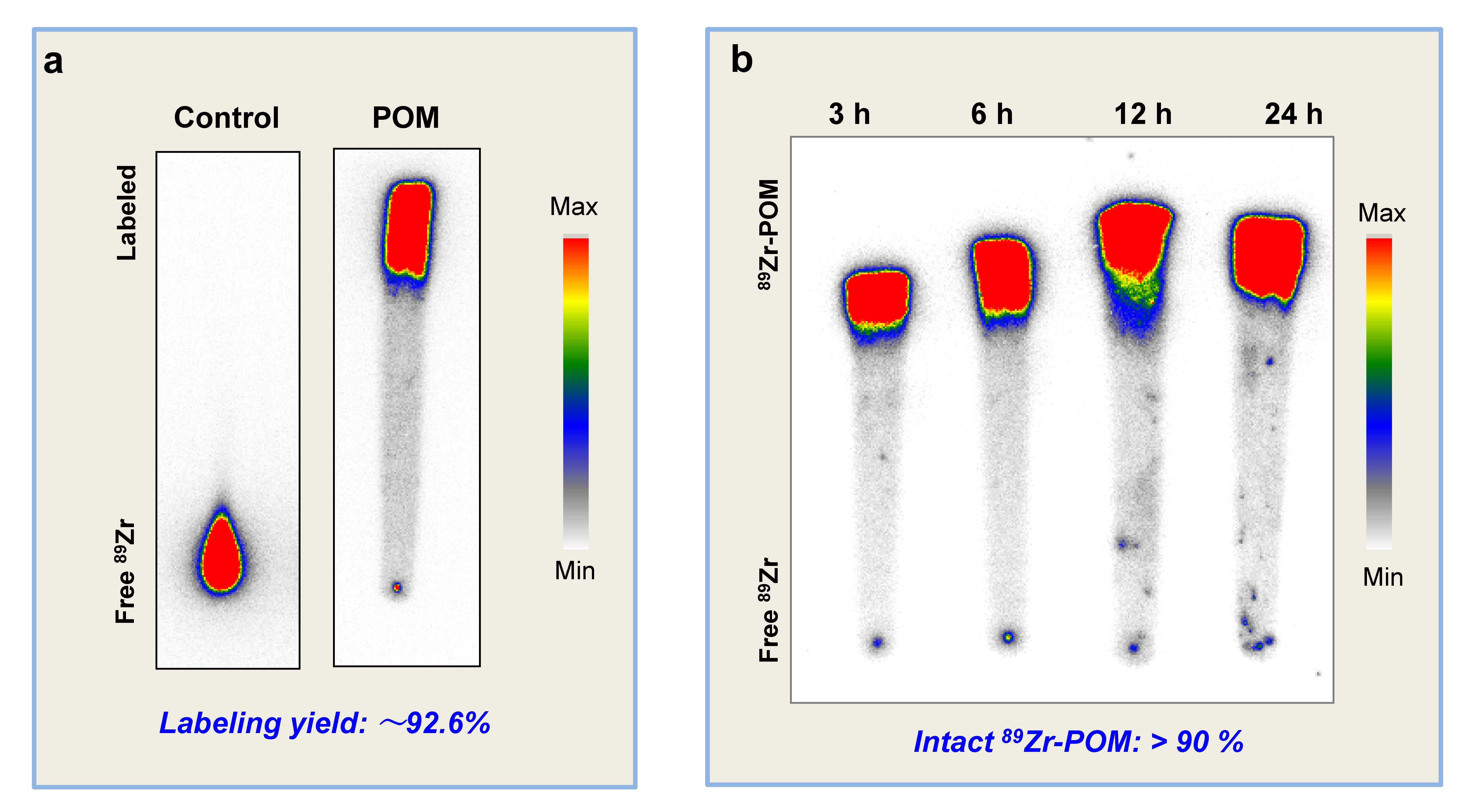


**Supplementary Figure 8.** Characterization of radiolabeling. **a,** The autoradiographic image of thin layer chromatography (TLC) plates of ^89^Zr-POM nanoclusters acquired at 37 °C using PBS (pH = 5) as the developing solvent. Free ^89^Zr-oxalate was used as a control. **b,** The stability of ^89^Zr-POM in PBS monitored by TLC after incubation at different time points.


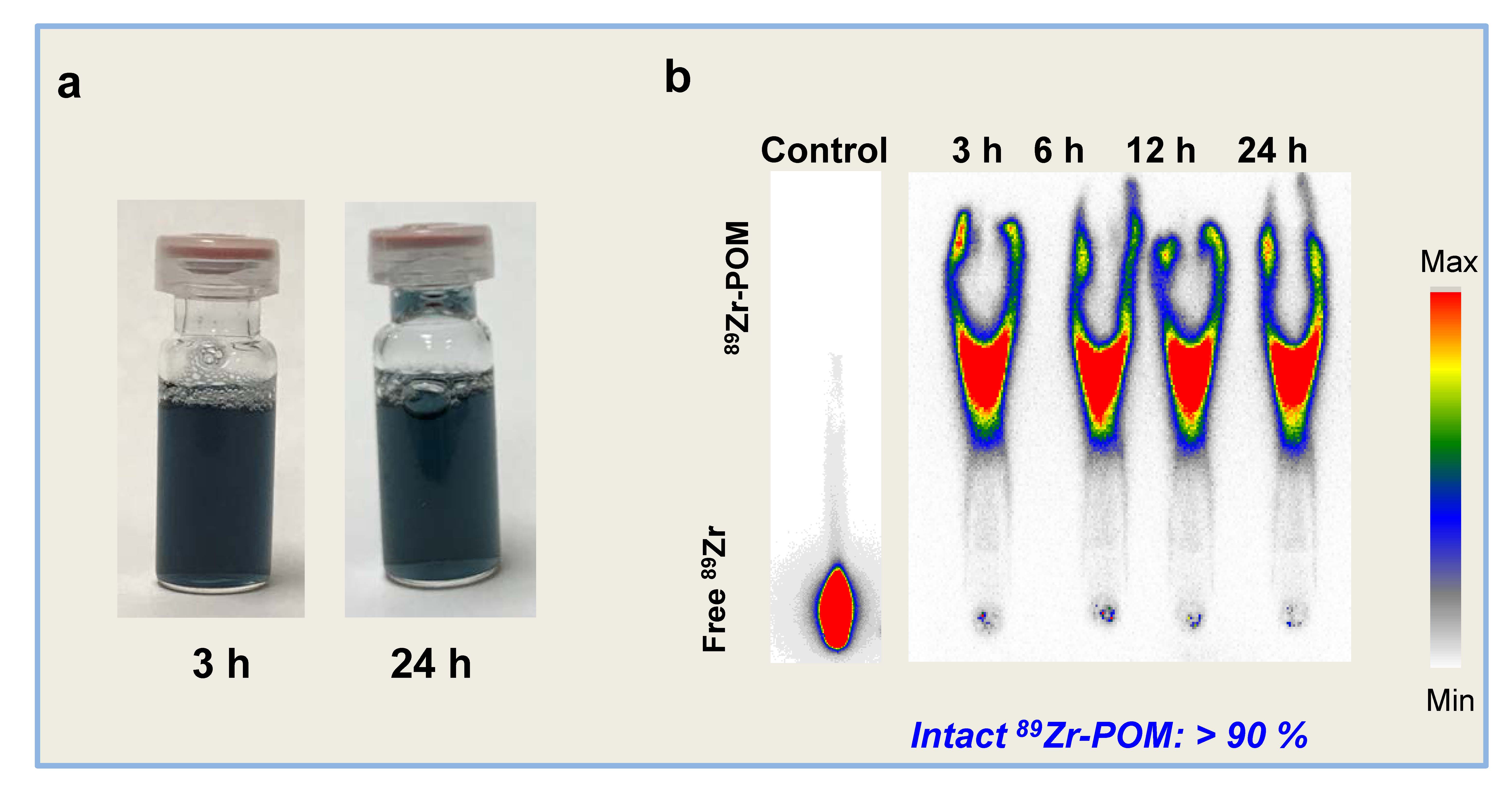


**Supplementary Figure 9**. Stability of POM nanoclusters. **a.** Photographs of POMs dispersed in the mouse serum. **b.** The autoradiographic image of TLC plates of ^89^Zr-POM nanoclusters dispersed in mouse serum using PBS (pH = 5) as the developing solvent. A small fraction of the mixture (15 μL) at various time points was collected and diluted by PBS for TLC plates.


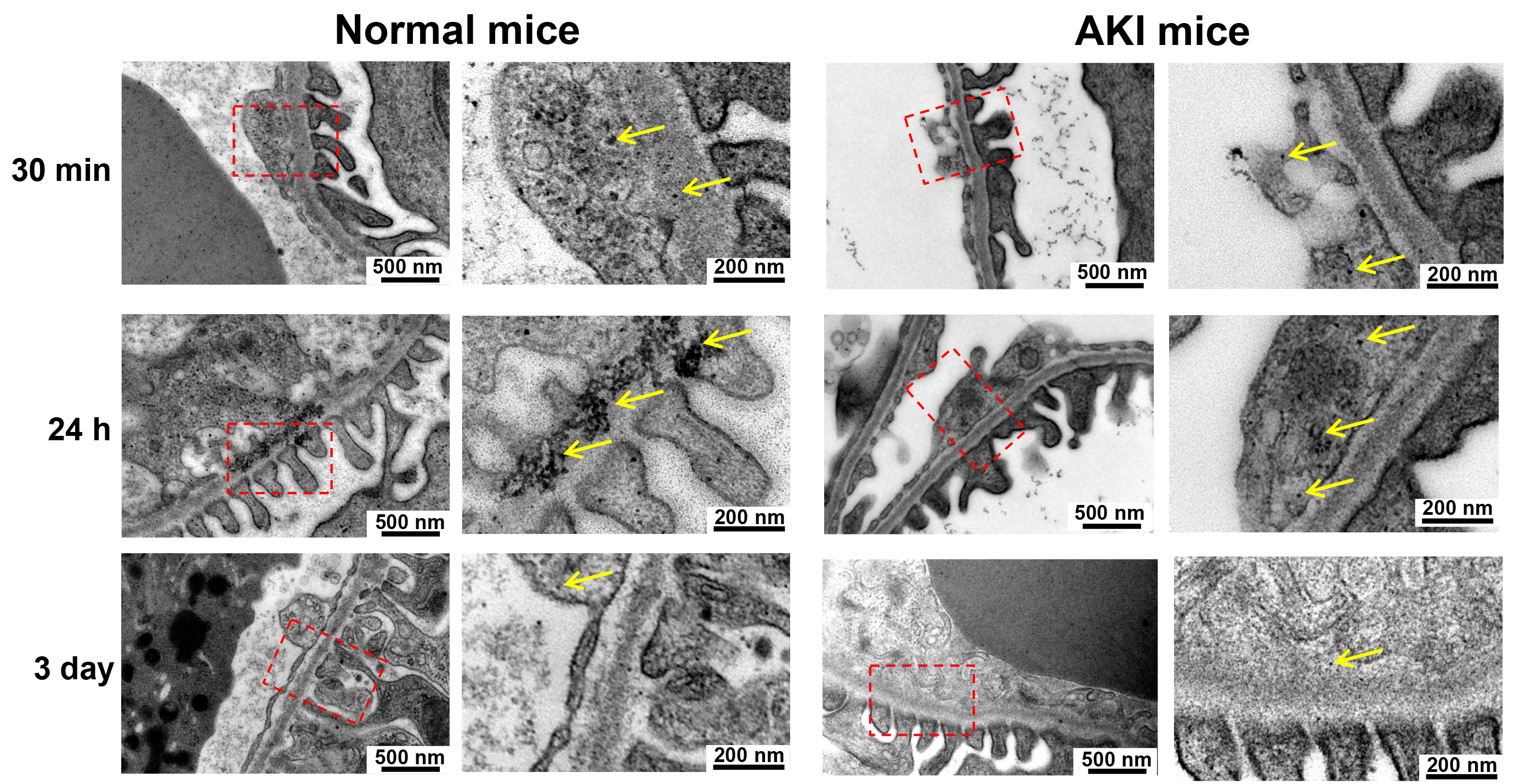


**Supplementary Figure 10.** Bio-TEM of renal tissues. Representative Bio-TEM images of the ultrastructure of the glomerular filtration membrane from healthy and AKI mice after intravenous injection of POM nanoclusters at 30 min, 24 h and 3 days. Yellow arrows indicated the presence of POM nanoclusters with some self-assembly.


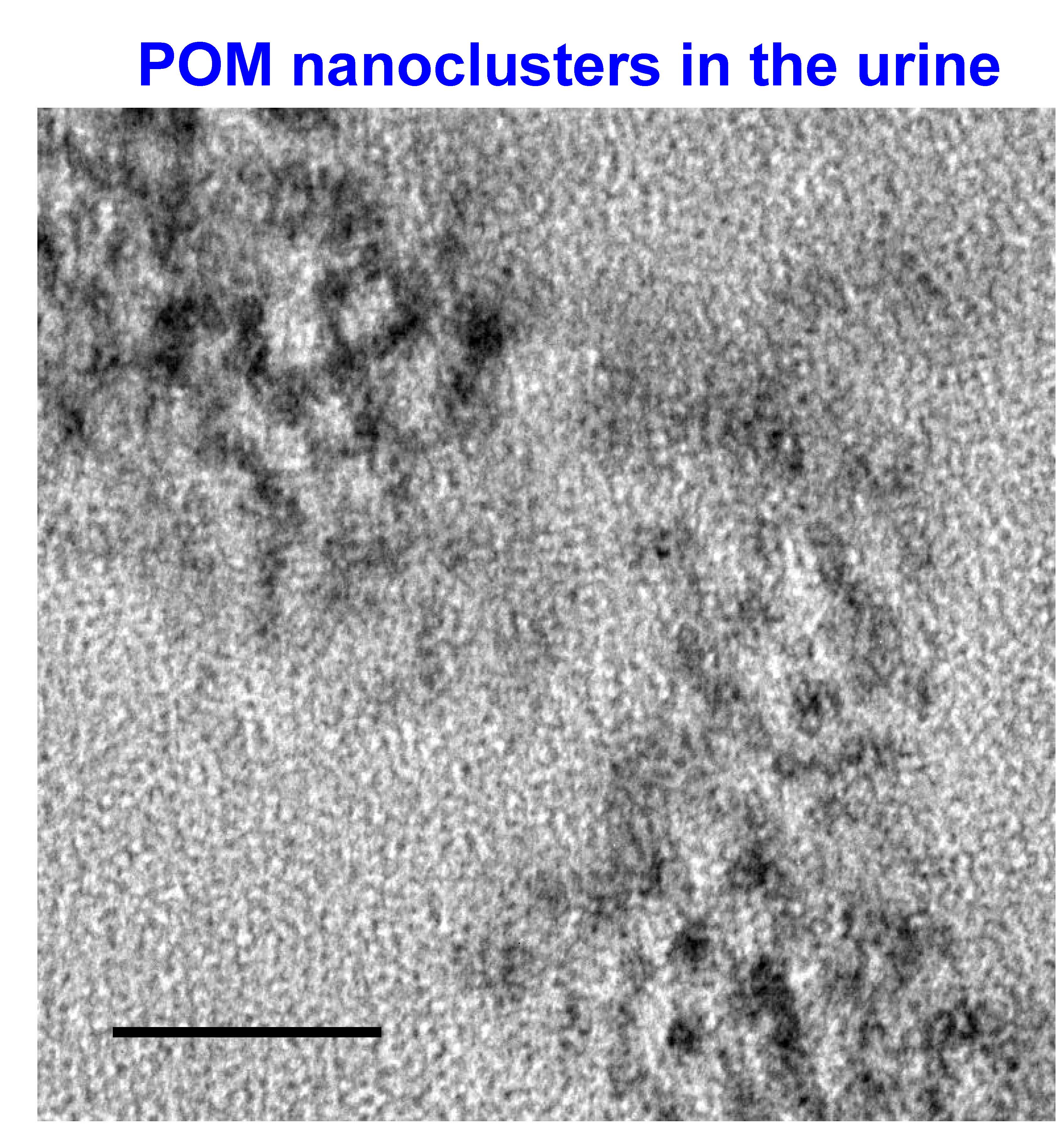


**Supplementary Figure 11.** POM nanoclusters in the urine. TEM image of the urine from the mouse after injection with the POM nanoclusters. Scale bar: 20 nm.

**Supplementary Figure 12.** Weight variation of AKI mice after treatment. Changes in body weight at 24 h after treatment of AKI with different conditions (n ≥ 4, mean ± s.d.).

**
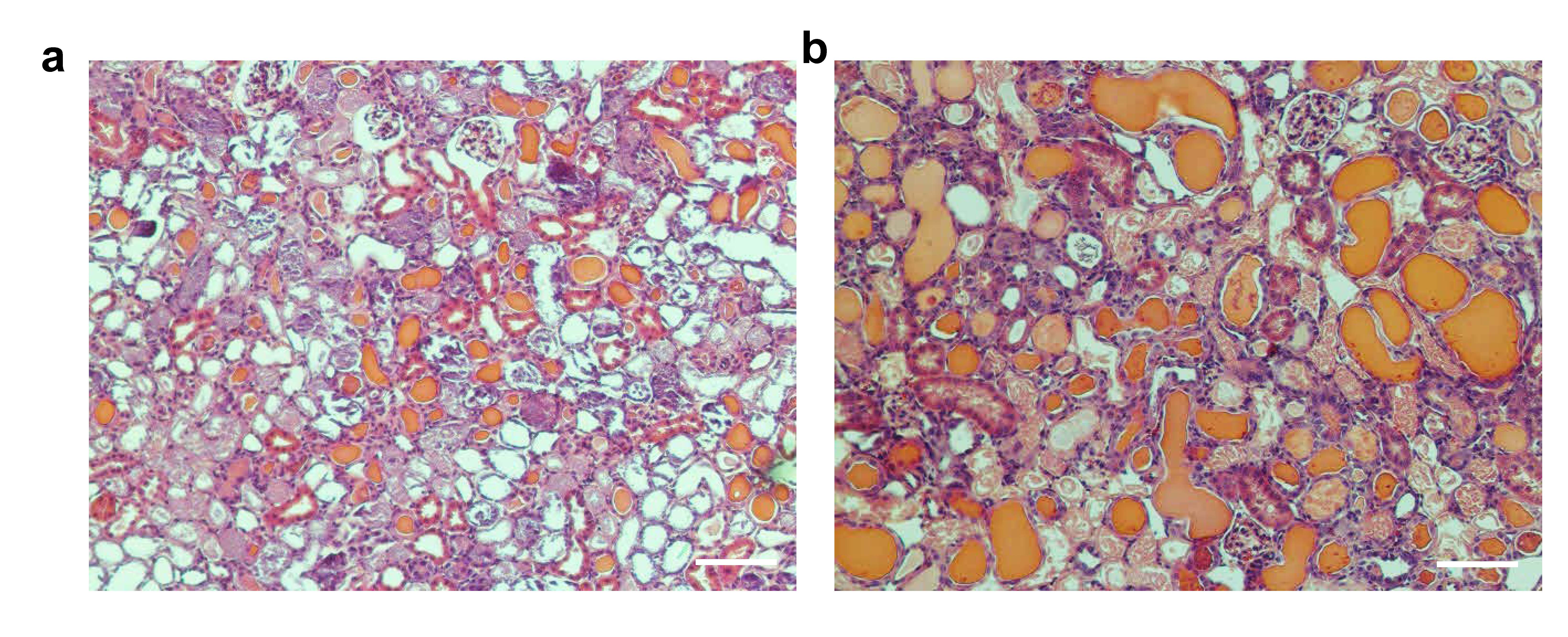
**

**Supplementary Figure 13.** Long-term assessment of AKI mice after PBS treatment. H&E-stained renal tissues from AKI group treated with PBS after 2 days **(a)** or died (**b)** with AKI after 3 days of treatment. Damaged tubules and the formation of casts were observed. Scale bar: 100 µm.


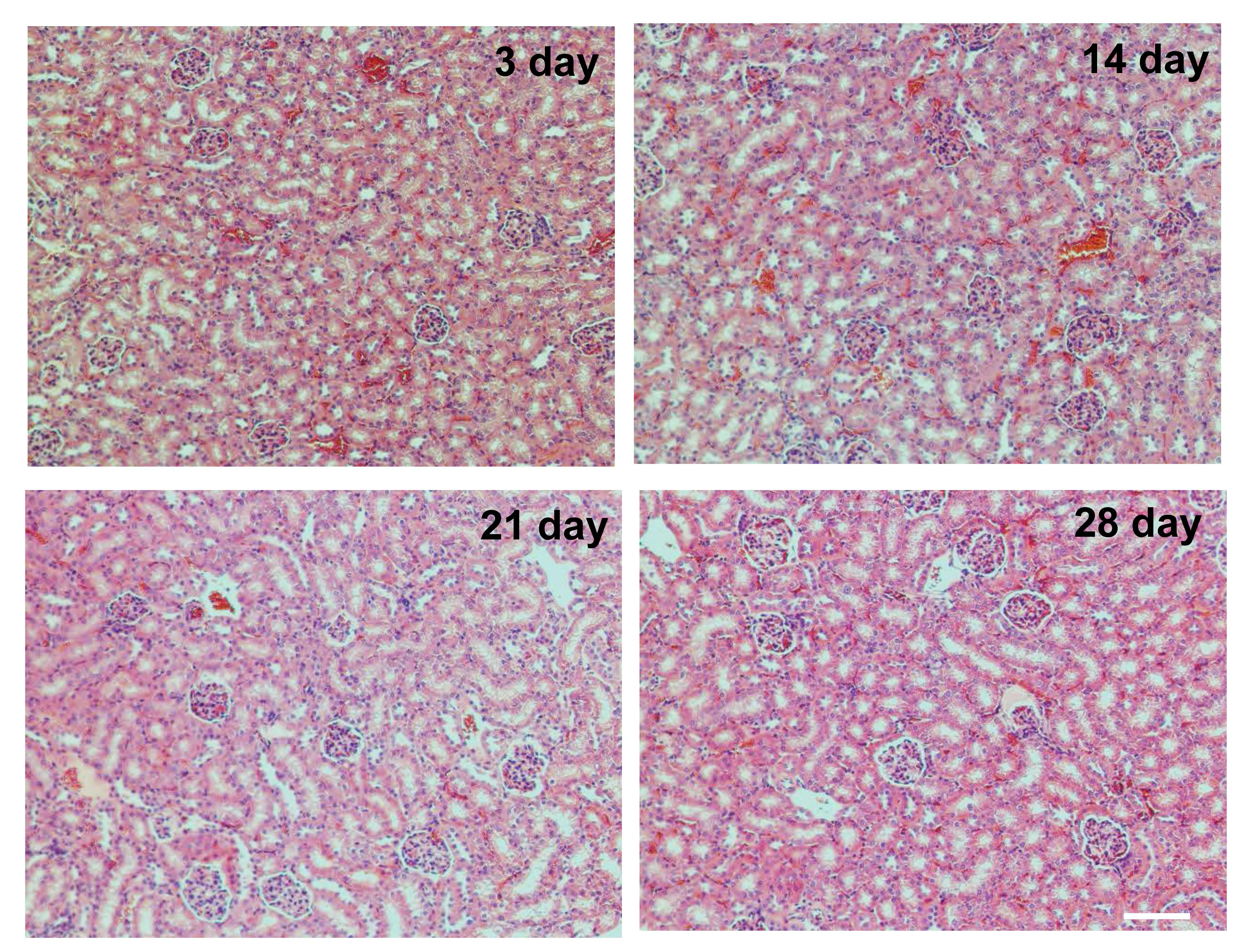


**Supplementary Figure 14.** Long-term assessment of AKI mice after POM treatment. H&E-stained renal tissues from AKI group treated with POM nanoclusters after 3, 14, 21, and 28 days of treatment. Scale bar: 100 µm.


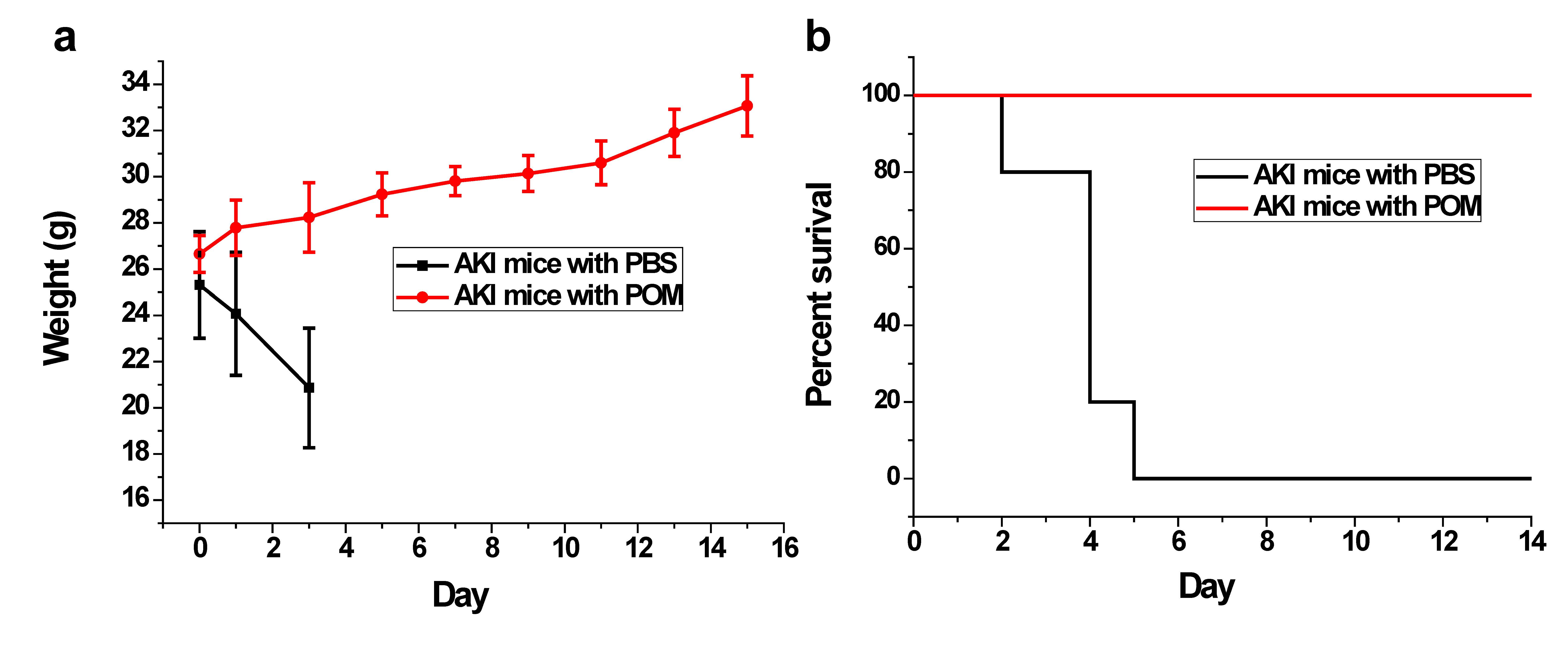


**Supplementary Figure 15.** Treatment of AKI. Changes in body weight (**a**) and survival curve (**b**) of AKI mice in the two weeks following PBS or POM treatment (n = 5, mean ± s.d.).


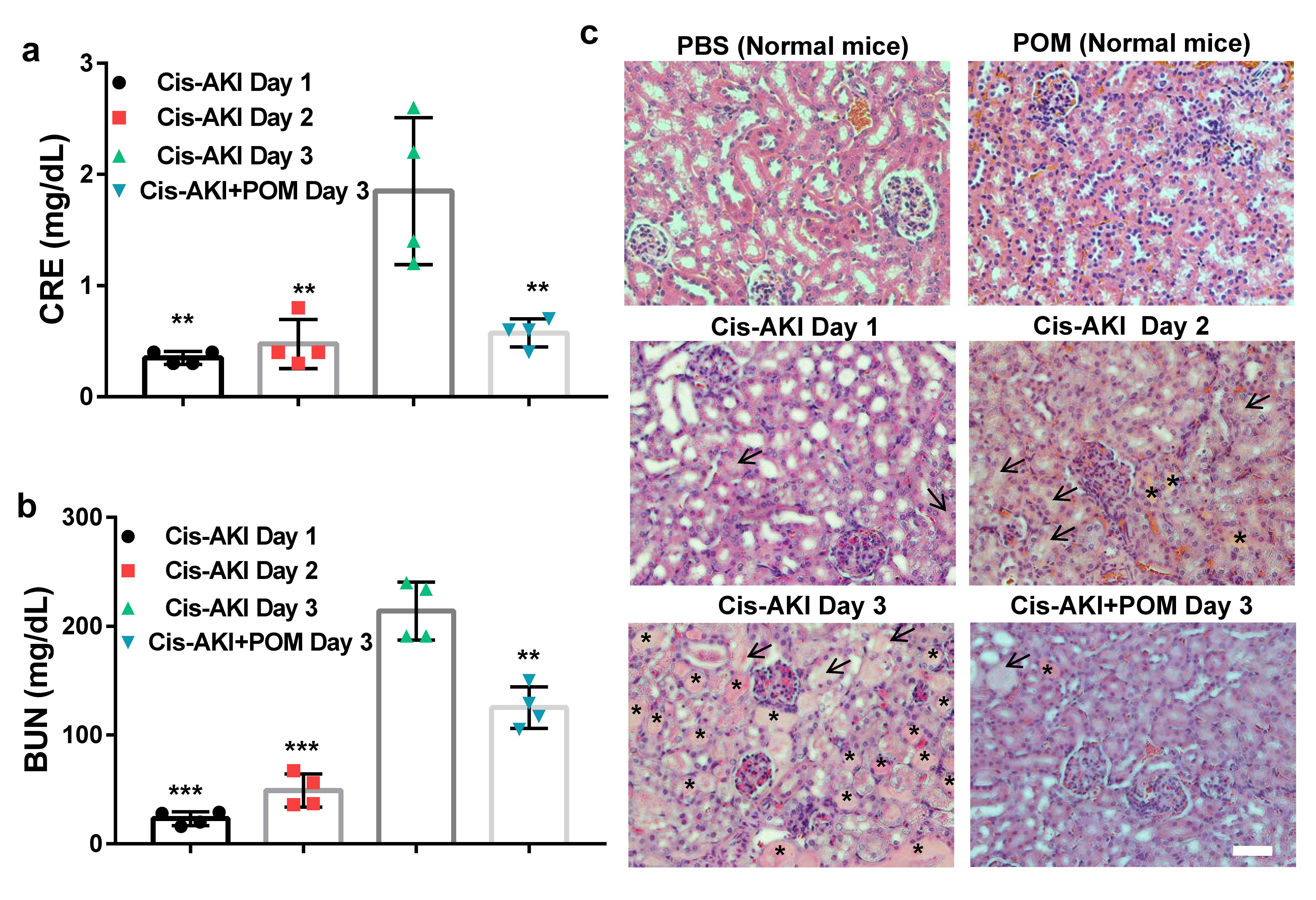


**Supplementary Figure 16.** Treatment of Cis-AKI. **a.** Creatinine (CRE) levels and **b.** blood urea nitrogen (BUN) in the blood serum from each group. Lower BUN and CRE levels denote better kidney functions. (n = 4; mean ± s.d.). *P* values were calculated by two-tailed Student’s t-test (** p < 0.01,*** p < 0.001). **c.** H&E staining of kidney tissues from each group. Arrows indicate damaged tubules, and asterisks indicate the formation of casts. Scale bar: 50 µm.

**
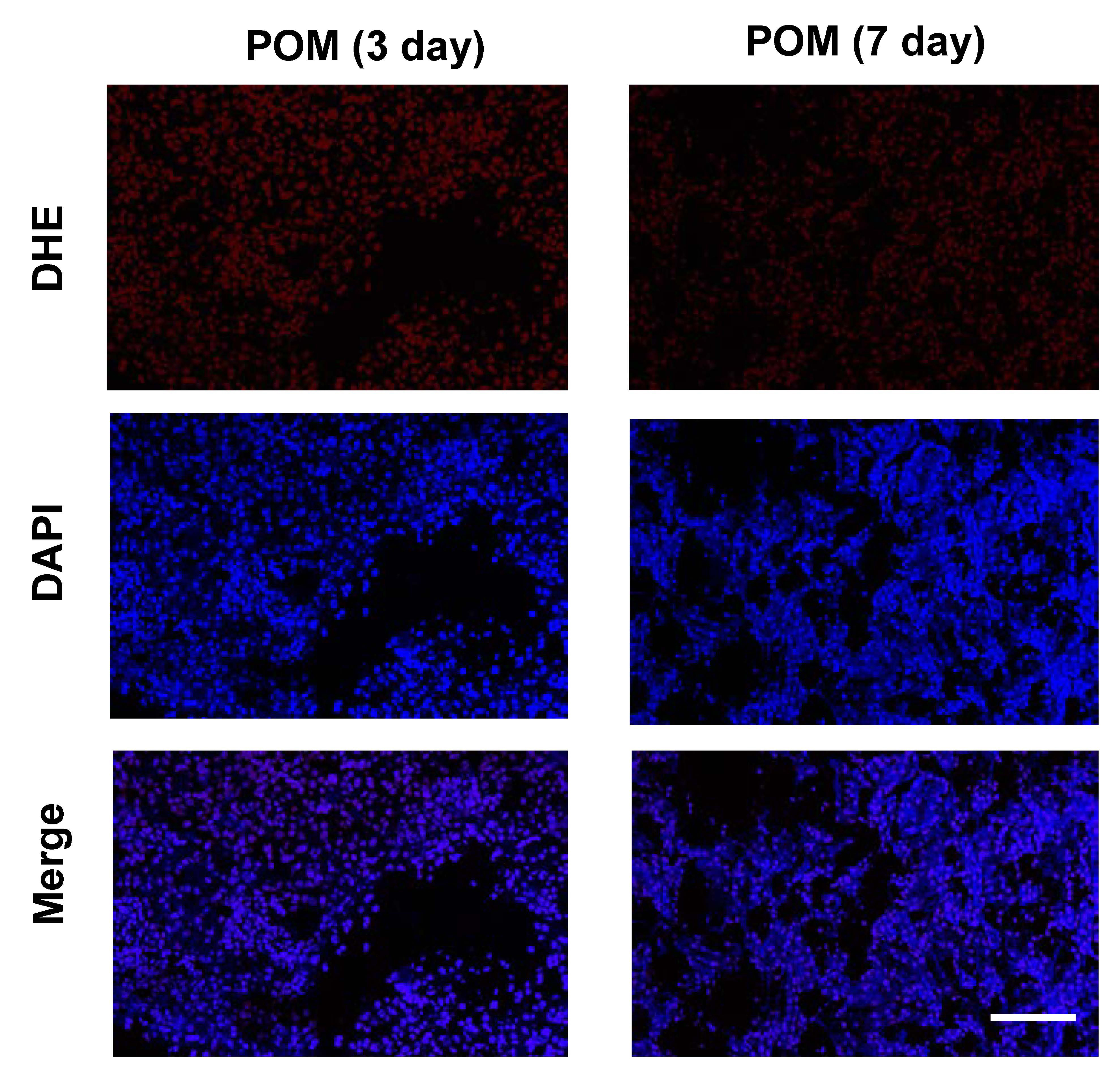
**

**Supplementary Figure 17.** Confocal imaging of renal tissues. DHE and DAPI staining of kidney tissues from mice at 3 days and 7 days p.i. Scale bar: 100 µm.

**
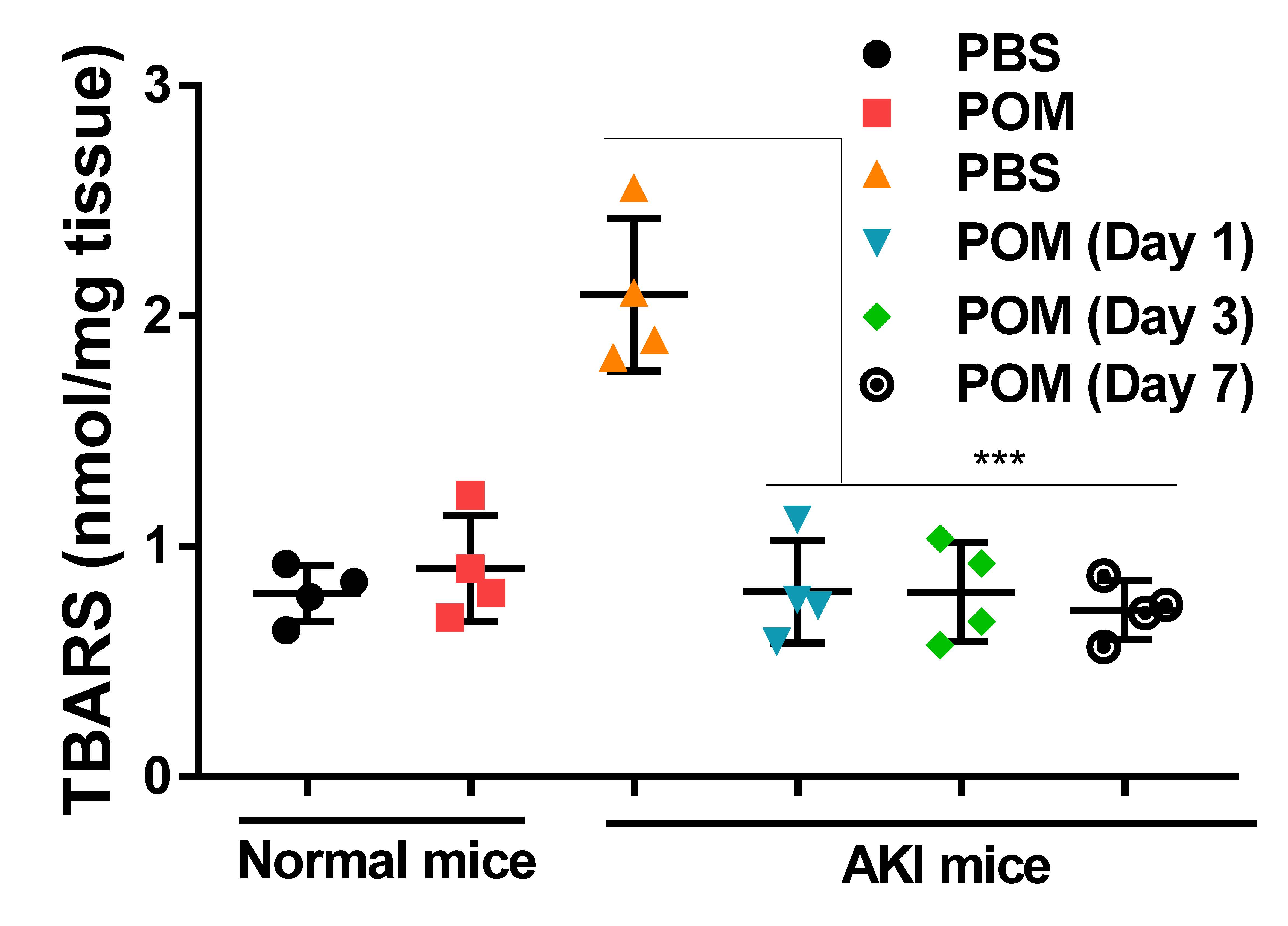
**

**Supplementary Figure 18.** Lipid peroxidation detection. Measurement of thiobarbituric acid-reactive substances (TBARS) in the kidneys of mice from each group. The TBARS levels served as an index of lipid peroxidation in the kidney (n = 4, mean ± s.d.). P values were calculated by two-tailed Student’s t-test (*** p < 0.001).


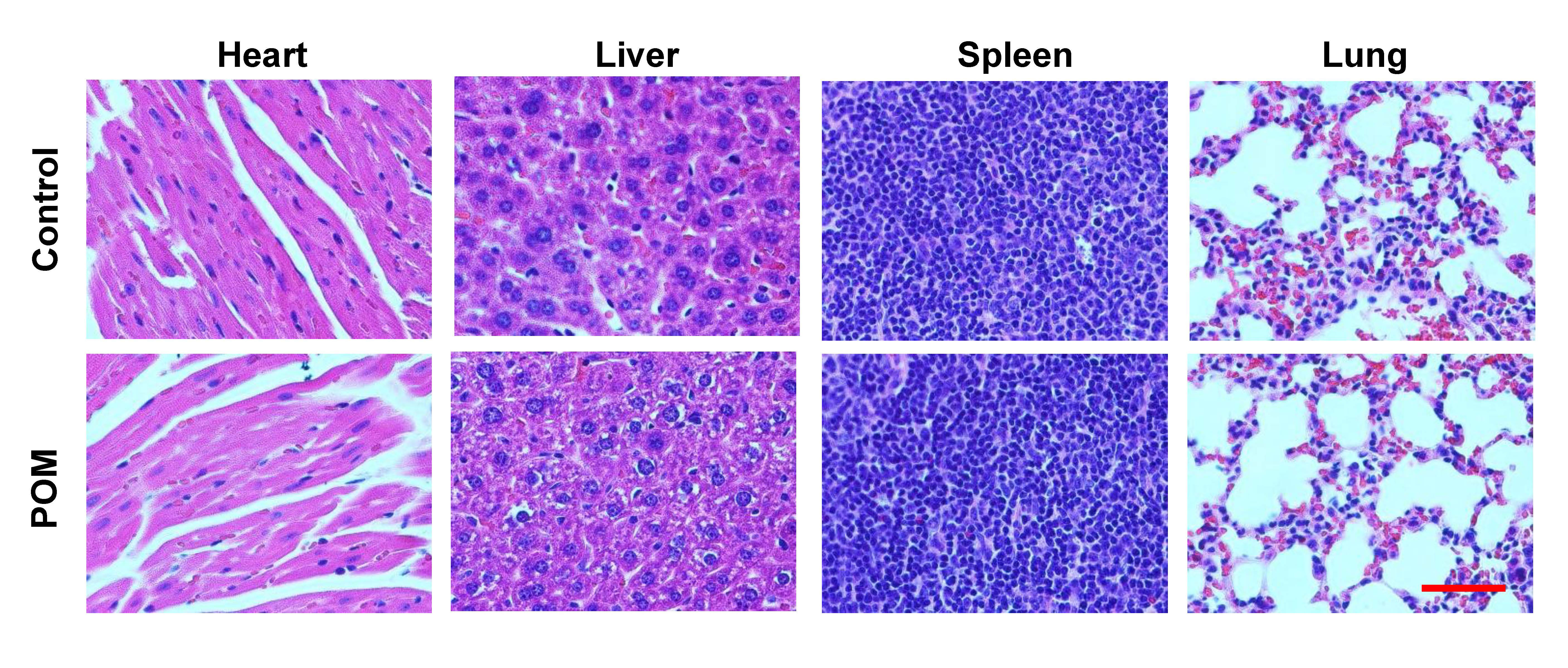


**Supplementary Figure 19.** *In vivo* toxicity assessment at 1 day p.i. H&E-stained tissues from mice to monitor the histological changes in the heart, liver, spleen, and lungs before and 1 day after intravenous injection of POM nanoclusters. Scale bar: 50 μm.

**
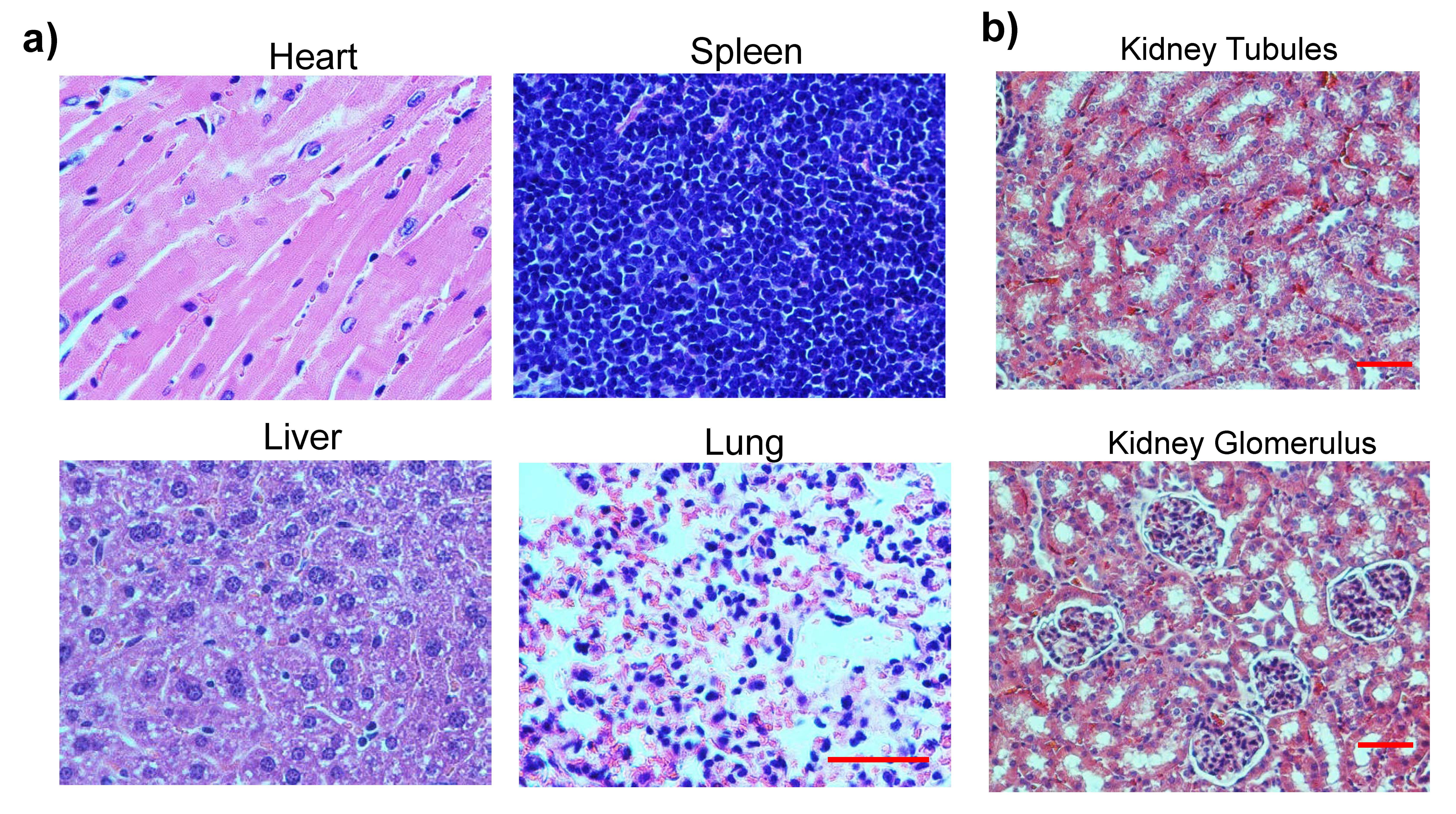
**

**Supplementary Figure 20.** *In vivo* toxicity assessment at 30 days p.i. H&E-stained tissues from mice to monitor the histological changes in a) heart, liver, spleen, lungs and b) kidneys at 30 days p.i. of POM clusters. Scale bar: 50 μm.
